# Metabolic regulation of 5-oxoproline for enhanced heat tolerance in perennial ryegrass

**DOI:** 10.1007/s44154-024-00175-9

**Published:** 2024-11-11

**Authors:** Shuhan Lei, Stephanie Rossi, Zhimin Yang, Jingjin Yu, Bingru Huang

**Affiliations:** 1 College of Agro-grassland Science, Nanjing Agricultural University, Nanjing, Jiangsu 210095 P.R. China; 2 Department of Plant Biology, Rutgers University, New Brunswick, NJ 08901 USA; 3 Institute of Environmental Processes and Pollution Control, School of Environmental and Ecology, Jiangnan University, Wuxi, 214122 P.R. China

**Keywords:** Heat stress, Pyroglutamic acid, Metabolism, Perennial ryegrass

## Abstract

Pyroglutamic acid [(5-oxoproline (5-oxp)], a non-protein amino acid, can be converted to glutamate to regulate amino acid metabolism in plants. Its roles in plant adaptation to abiotic stresses, including heat stress, are not well understood. The objectives of this study were to determine whether exogenous application of 5-oxp could promote heat tolerance in cool-season perennial grass species and identify the major metabolic pathways that could be activated or responsive to 5-oxp for enhancing heat tolerance. Perennial ryegrass (*Lolium perenne* L.) plants were foliar-sprayed with 5-oxp or water (untreated control) prior to and during the exposure to heat stress (35/33 ℃, day/night temperature) or ambient temperature (25/22 ℃, day/night temperature, non-stress control) in controlled-environment growth chambers. Application of 5-oxp improved the heat tolerance of perennial ryegrass, as manifested by the chlorophyll content, photochemical efficiency, cell membrane stability, and antioxidant enzyme activities increasing by 31.2%, 25.7%, 37.2%, and 57.1-258.3%, as well as the reduction in hydrogen peroxide production by 36.8%. Metabolic profiling identified metabolites up-regulated by 5-oxp that are involved in the metabolic pathways of carbon assimilation in photosynthesis, glycolysis and the tricarboxylic acid cycle of respiration, proteinogenic amino acid metabolism, glutathione metabolism, and nucleotide metabolism for DNA or RNA synthesis and ATP generation. The up-regulation or activation of those metabolic processes could contribute to 5-oxp-mediated enhancement in the heat tolerance of perennial ryegrass.

**Supplementary Information:**

The online version contains supplementary material available at 10.1007/s44154-024-00175-9.

## Introduction

With global warming, heat stress has become one of the major abiotic stresses limiting plant growth and productivity. Various approaches have been developed to mitigate heat injuries and improve plant tolerance to heat stress, including chemical priming (Francesca et al. 2020). Exogenous application of natural metabolites, including amino acids, has been widely used as a strategy to activate plant stress defense and metabolism against abiotic stresses in various plant species (Botta 2013).

Pyroglutamic acid, or 5-oxoproline (5-oxp), is a naturally occurring metabolite of poor functional characterization that is produced in the glutathione (GSH) cycle and converted into glutamate by 5-oxoprolinase in plant tissues (Ohkama-Ohtsu et al. 2008). The increase in endogenous 5-oxp content has been positively associated with tolerance to abiotic stresses, such as salt and heat stress in several perennial grass species, including creeping bentgrass (*Agrostis stolonifera* L.), perennial ryegrass (*Lolium perenne* L.), and tall fescue (*Festuca arundinacea* Schreb.) (Lei et al. 2021, Yang et al. 2014, Yu et al. 2012). The application of 5-oxp as a plant biostimulant resulted in increases in the yield of lettuce under drought stress by promoting antioxidant capacity (Jiménez-Arias et al. 2019). The roles or mechanisms of 5-oxp associated with the regulation of plant tolerance to heat stress are not well understood, despite the importance of this natural metabolite, which is involved in key metabolic processes for stress defense.

The objectives of this study were to investigate whether exogenous application of 5-oxp could promote heat tolerance of temperate perennial grasses and determine the major metabolic pathways that may be activated or responsive to 5-oxp for enhancing heat tolerance. Perennial ryegrass is a temperate grass species widely used as forage or turfgrass, but it is sensitive to heat stress (Zhang et al. 2020). Understanding the regulatory functions of 5-oxp is of great significance for developing heat-tolerant grass species through genetic manipulation or using it as a natural biostimulant to improve plant tolerance to heat stress associated with global warming.

## Results

### Physiological and antioxidative responses to 5-oxp application for perennial ryegrass under non-stress and heat stress conditions

**Fig. 1:**
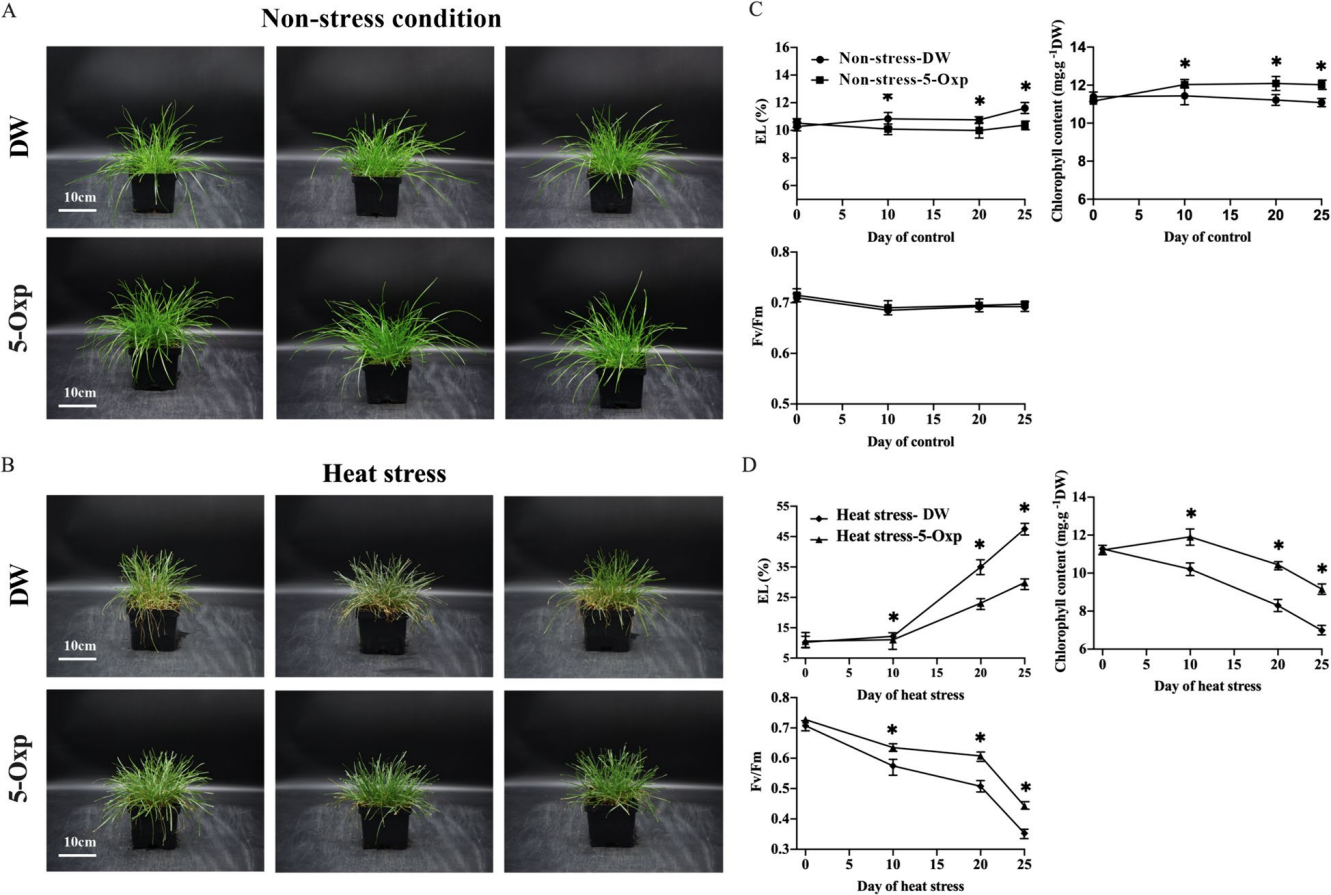
Phenotypes of enhancing heat resistance in perennial ryegrass regulated by DW and 5 mM 5-oxp at 25 days of non-heat stress **A** and heat stress **B**; effect of DW and 5-oxp on EL, Chl, and F_v_/F_m_ in perennial ryegrass leaves under 25 days of non-heat stress **C** and heat stress **D**. White bars in phenotype are in 10 cm. Vertical bars stand for standard errors of a certain data point. *: significant differences between DW and 5-oxp on a certain day of control or heat stress treatment based on the LSD test at *p* = 0.05 (the same below)

Plants treated with 5-oxp demonstrated more vigorous turf growth and greener leaves in comparison to untreated plants under heat stress, as demonstrated in Fig. 1A and B, which depict the phenotypes of 5-oxp-treated plants and untreated plants exposed to non-heat stress conditions or heat stress. Under non-stress conditions, 5-oxp-treated plants had significantly lower leaf electrolyte leakage (EL) and higher chlorophyll (Chl) content in comparison to untreated plants from 10–25 days, but their photochemical efficiency (F_v_/F_m_) was not significantly different (Fig. 1C).

Leaf EL significantly increased while Chl content and F_v_/F_m_ significantly decreased in all treated plants under a 25-day heat stress period; however, from 10–25 days of heat stress, 5-oxp-treated plants maintained significantly lower leaf EL and ignificantly higher Chl content and F_v_/F_m_ than untreated plants (Fig. 1D).

**Fig. 2:**
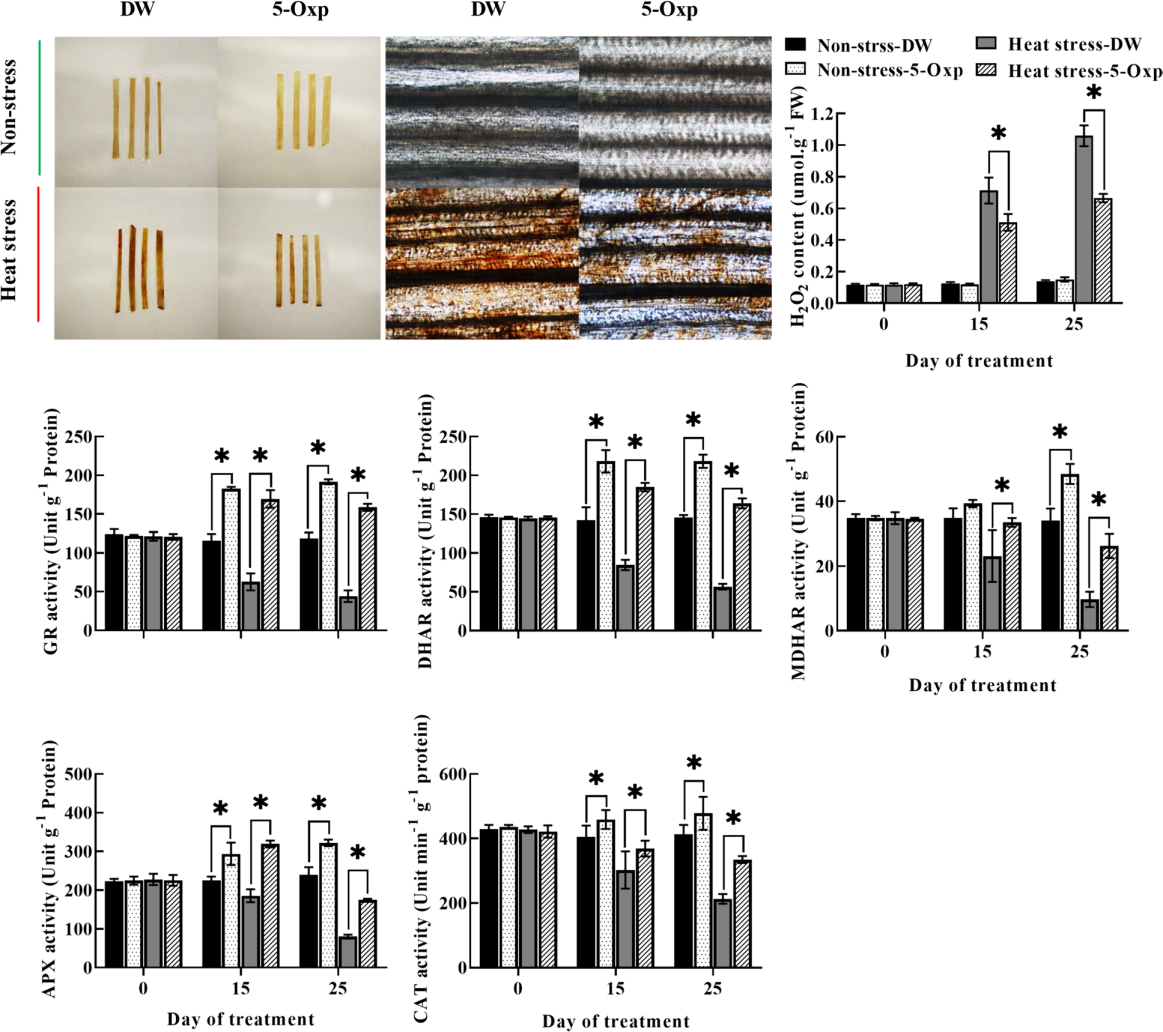
Impact of 5 mM exogenous 5-oxp on anti-oxidation of perennial ryegrass under heat stress; **A** phenotype of DAB chemical dyeing and H_2_O_2_ content in perennial ryegrass leaves treated with DW and 5-oxp at 25 days of non-heat stress or heat stress; **B** activity of antioxidant enzymes controlled by DW and 5-oxp in perennial ryegrass leaves under 25 days of non-heat stress or heat stress. Vertical bars over the column are standard errors of a certain data point

Leaf hydrogen peroxide (H_2_O_2_) accumulation in 5-oxp-treated or untreated plants was not different under non-stress conditions. From 15–25 days of heat stress, H_2_O_2_ content in 5-oxp-treated plants was significantly lower than that of untreated plants (Fig. 2A). The activities of glutathione reductase (GR), dehydroascorbate reductase (DHAR), monodehydroascorbate reductase (MDHAR), catalase (CAT), and ascorbate peroxidase (APX) in 5-oxp-treated plants were significantly higher than those in the untreated plants exposed to heat stress from 15–25 days (Fig. 2B).

## Metabolic responses of perennial ryegrass to 5-oxp under non-stress and heat stress conditions

**Fig. 3:**
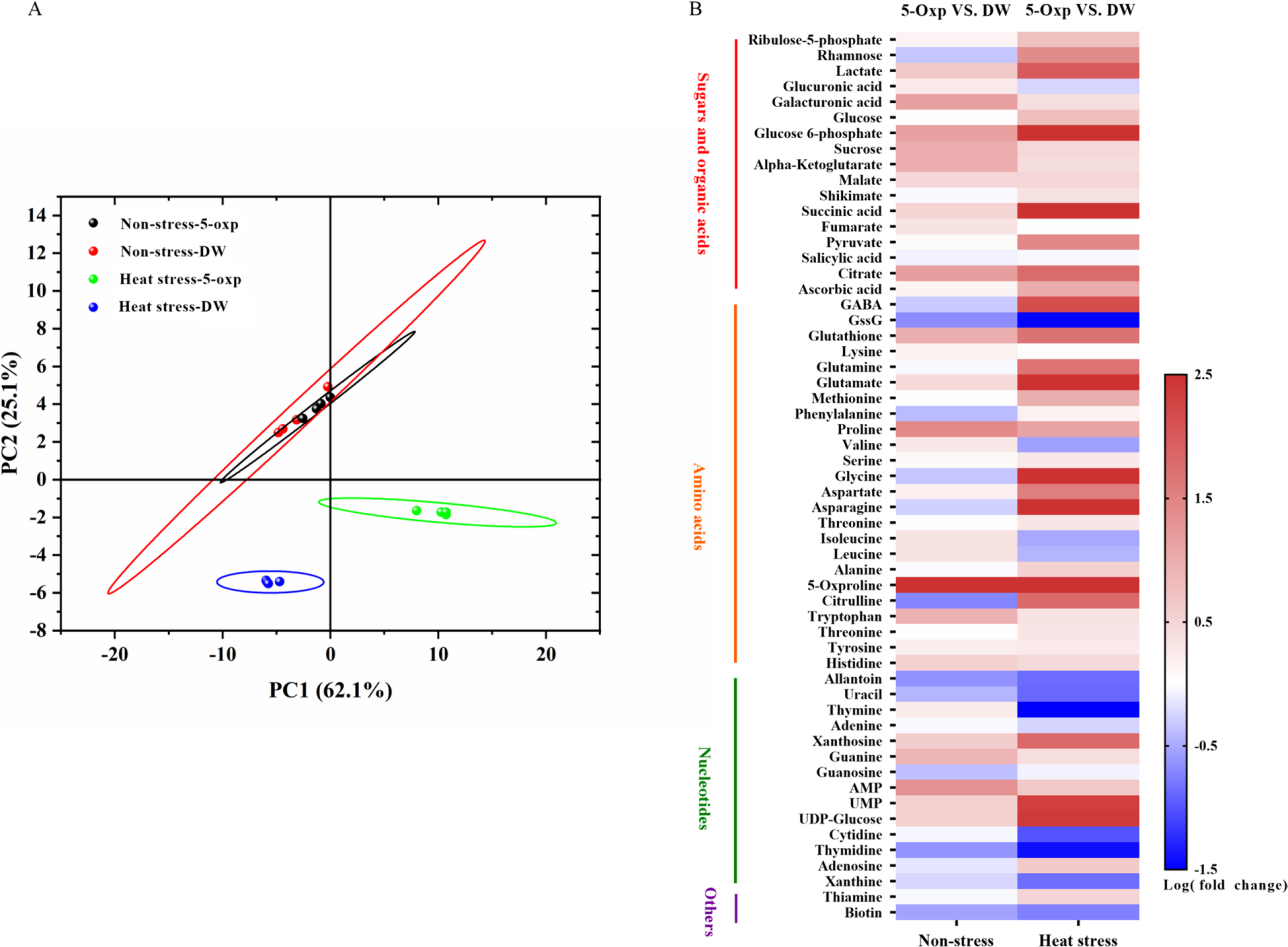
PCA of differentially regulated metabolites by DW- and 5 mM 5-oxp- treated perennial ryegrass under 25 days of non-heat stress or heat stress **A**; Heat map of fold-changes for metabolites responsive to 5 mM 5-oxp with up-regulation (red bars) or down-regulation (blue bars) in metabolite contents after 5-oxp treatment versus DW treatment under 25 days of non-heat stress or heat stress **B**

To determine key metabolites and metabolic pathways regulated by 5-oxp that could be linked to an improvement in heat tolerance, metabolomics analysis was performed. The first principal component analysis (PCA) shown consisted of metabolites of untreated and 5-oxp-treated plants (62.1%), distinctly responsive to 5-oxp-treatment in comparison to those of the untreated control under non-stress or heat stress conditions, which were not responsive. The temperature treatments constituted the second PCA (25.1%), which exhibited a distinct separation of metabolites affected by heat stress from those of the non-stress controls (Fig. 3A).

**Fig. 4:**
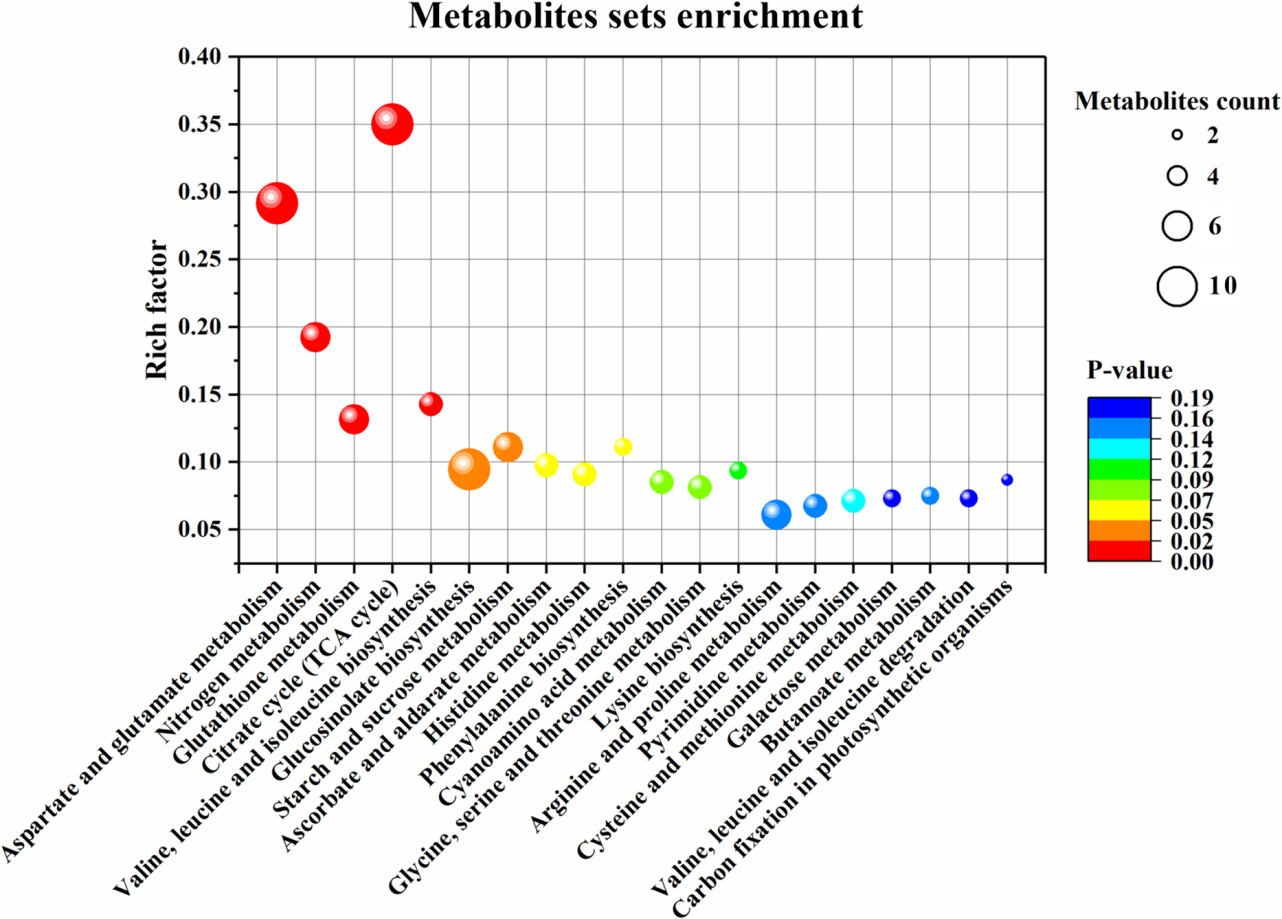
Metabolic pathways enrichment analysis of 38 significantly differential metabolites by 5 mM 5-oxp in perennial ryegrass leaves at 25 days of heat stress. The bubble size represents the number of 5-oxp-responsive metabolites enriched in each metabolic pathway. The color of bubbles represents the level of significance based on the LSD test (*p* = 0.05)

Fifty-seven metabolites that were responsive to 5-oxp in perennial ryegrass were identified under both non-stress and heat stress conditions through a heat map (Fig. 3B and Table 1). Among the 57 metabolites, the content of 38 metabolites was significantly different between 5-oxp-treated and untreated plants under non-stress or heat stress conditions, and these 38 metabolites were mainly enriched in the pathways of (1) aspartate and glutamate metabolism, (2) nitrogen metabolism, (3) glutathione metabolism, (4) citrate cycle, (5) valine, leucine, and isoleucine biosynthesis, as well as (6) starch and sucrose metabolism (Fig. 4).

**Table 1:** Response of metabolites to 5-Oxoproline, regulating heat tolerance in perennial ryegrass

No.	RT(min)	Metabolite	Mz	No.	RT(min)	Metabolite	Mz
1	3.36	Rhamnose	163.06	30	6.48	Alanine	88.04
2	12.94	Ribose-5-phosphate	232.08	31	7.01	Glycine	74.02
3	9.60	UDP-Glucose	565.05	32	7.49	Serine	104.04
4	8.23	Glucuronic acid	193.04	33	7.45	GABA	102.06
5	8.23	Galacturonic acid	193.04	34	6.76	Threonine	118.05
6	1.99	Glucuronolactone	175.03	35	4.33	Isoleucine	130.09
7	10.58	Glucose 6-phosphate	259.02	36	3.88	Leucine	130.09
8	7.14	Sucrose	341.11	37	4.73	Pyroglutamate	128.04
9	5.77	Glucose	179.06	38	4.94	Tyrosine	180.07
10	8.27	Myo-Inositol	179.06	39	4.39	Tryptophan	203.08
11	2.54	Pyruvate	87.01	40	9.17	Histidine	154.06
12	11.51	Citrate	191.02	41	3.38	Guanine	150.04
13	7.34	Alpha-Ketoglutarate	145.01	42	3.04	Allantoin	157.04
14	8.79	Malate	133.01	43	2.12	Uracil	111.02
15	7.55	Fumarate	115.00	44	2.66	Adenine	134.05
16	8.28	Succinate	117.02	45	1.99	Thymine	125.04
17	7.05	Shikimate	173.05	46	4.17	Guanosine	282.08
18	3.87	Lactate	89.02	47	3.92	Xanthine	151.03
19	1.36	Salicylic acid	137.02	48	4.97	Xanthosine	283.07
20	5.73	Ascorbic acid	175.03	49	9.40	AMP	346.06
21	12.63	Lysine	145.10	50	9.46	UMP	323.03
22	7.30	Glutamine	145.06	51	3.90	Cytidine	242.08
23	8.48	Glutamate	146.05	52	2.06	Thymidine	241.08
24	4.45	Methionine	148.04	53	2.64	Adenosine	266.09
25	8.70	Aspartate	132.03	54	6.15	Thiamine	263.10
26	7.54	Asparagine	131.05	55	12.25	GSSG	611.15
27	3.51	Phenylalanine	164.07	56	8.71	Glutathione	306.08
28	5.85	Proline	114.06	57	5.16	Biotin	243.08
29	5.38	Valine	115.00

**Fig. 5:**
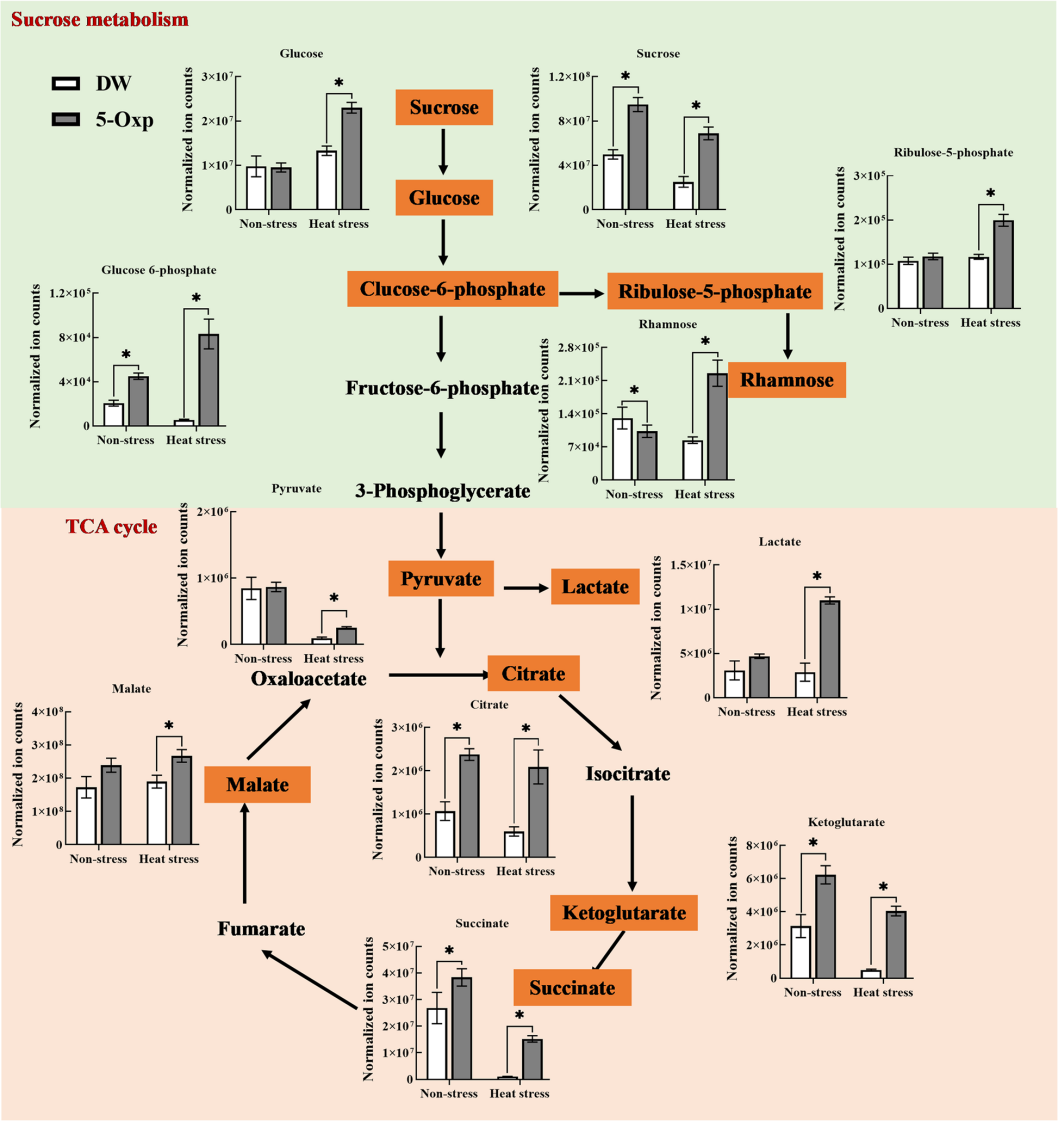
The effects of exogenous application of 5-oxp (5 mM) on the sucrose metabolism and TCA cycle in leaves of perennial ryegrass under 25 days of non-stress and heat stress

Several sugars involved in glycolysis, including sucrose, glucose, glucose 6-phosphate, ribulose 5-phosphate, and rhamnose, were up-regulated by 5-oxp in plants, as the content of those metabolites was significantly higher in 5-oxp-treated plants than in untreated plants, particularly for glucose, glucose 6-phosphate, ribulose 5-phosphate, and rhamnose under heat stress (Fig. 5). The content of six organic acids in the tricarboxylic acid (TCA) cycle (lactate, pyruvate, citrate, ketoglutarate, succinate, and malate) also increased due to the application of 5-oxp, and pyruvate, malate, lactate, and succinate was elevated to a greater extent in plants exposed to heat stress (Fig. 5).

**Fig. 6:**
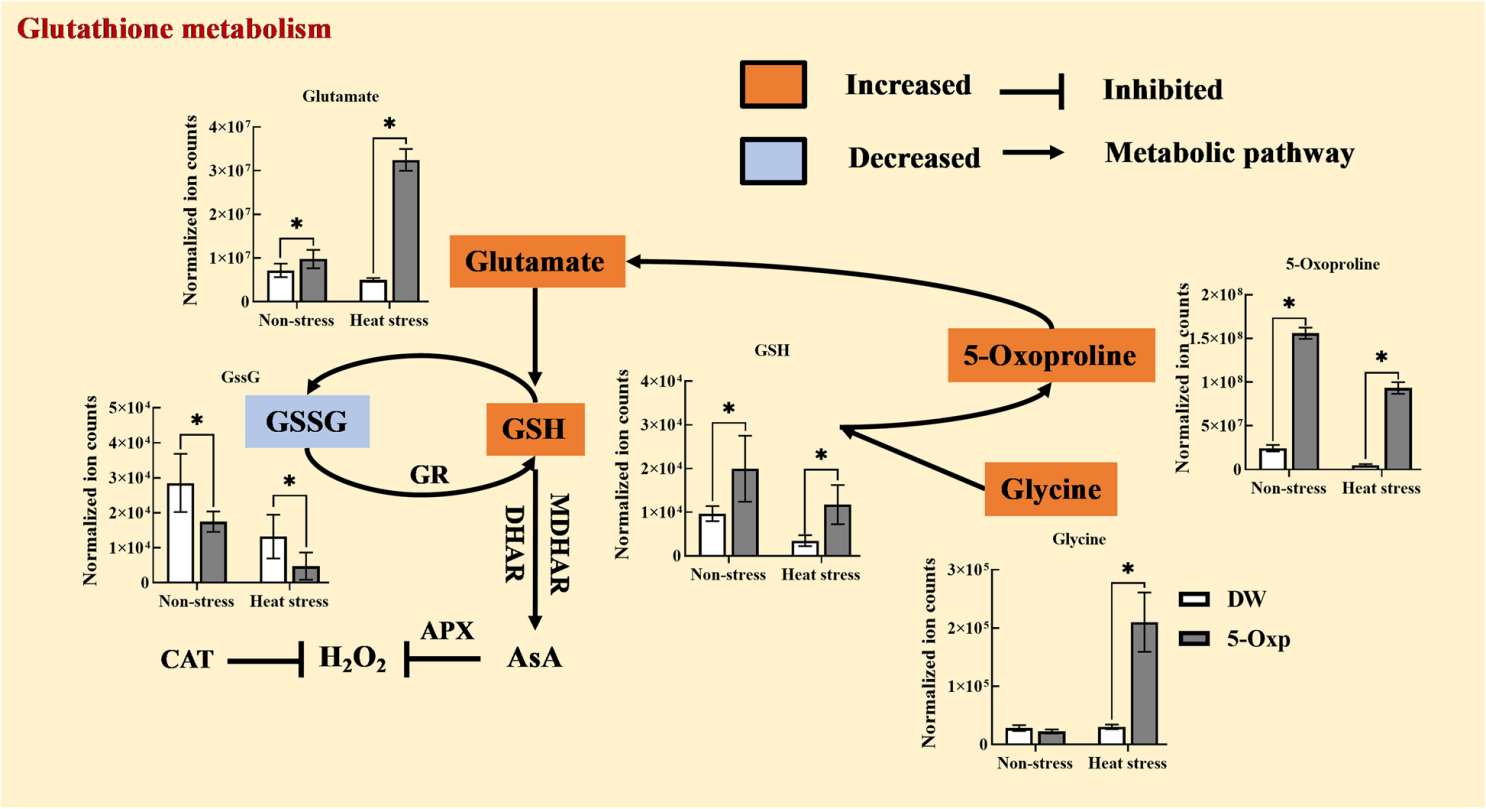
The effects of exogenous application of 5-oxp (5 mM) on the glutathione metabolism in leaves of perennial ryegrass under 25 days of non-stress and heat stress. The black arrows and black vertical lines represent promotion and inhibition, respectively, and orange and blue represent significantly up-regulated and down-regulated metabolites by 5-oxp treament compared with DW treatment under heat stress based on the LSD test (*p* = 0.05) (the same below)

**Fig. 7:**
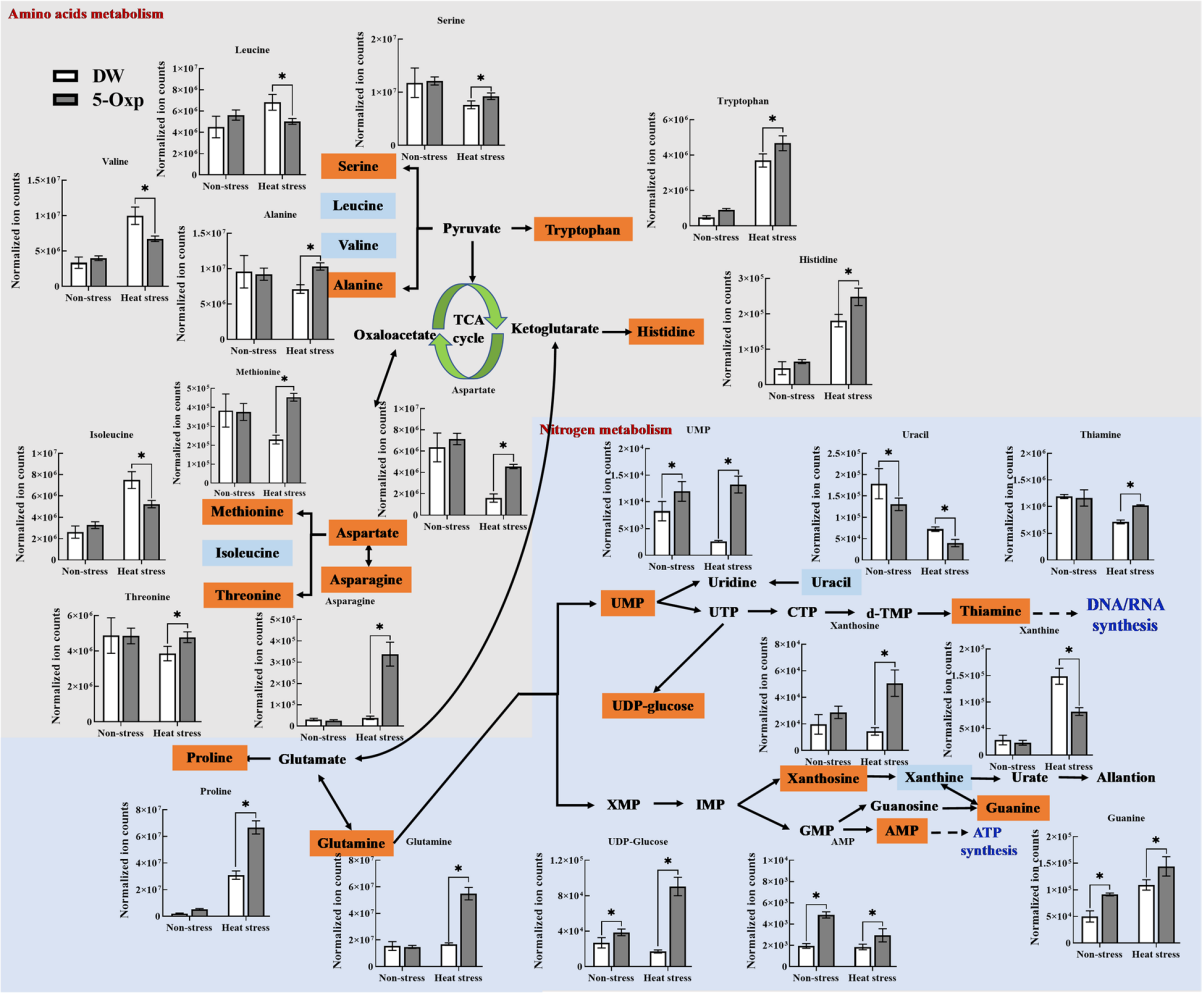
The effects of exogenous application of 5-oxp (5 mM) on the amino acids and nitrogen metabolism in leaves of perennial ryegrass under 25 days of non-stress and heat stress

In glutathione metabolism, the endogenous content of glutamate, glycine, 5-oxp, and glutathione (GSH) was significantly increased with the application of 5-oxp under non-stress and heat stress conditions (Fig. 6). The up-regulation of glutamate and glycine in response to 5-oxp was more pronounced under heat stress relative to their responses to 5-oxp under non-stress conditions. Ten other amino acids (serine, tryptophan, alanine, histidine, methionine, aspartate, asparagine, threonine, proline, and glutamine) exhibited an up-regulation in response to 5-oxp, as they had significantly higher content in 5-oxp-treated plants than in untreated plants only under heat stress (Fig. 7).

Five nucleotides [uridine monophosphate (UMP), uridine diphosphate-glucose (UDP-glucose), xanthosine, guanine, and adenosine monophosphate (AMP)] had significantly increased content in 5-oxp-treated plants compared to untreated plants under non-stress or heat stress conditions (Fig. 7). The elevation was most pronounced in UMP, UDP-glucose, and xanthosine, while thiamine was up-regulated by 5-oxp only under heat stress. The content of three amino acids (leucine, valine, and isoleucine) and two nucleotides (uracil and xanthine) decreased significantly in 5-oxp-treated plants with respect to those of untreated plants under heat stress (Fig. 7).

## Discussion

**Fig. 8:**
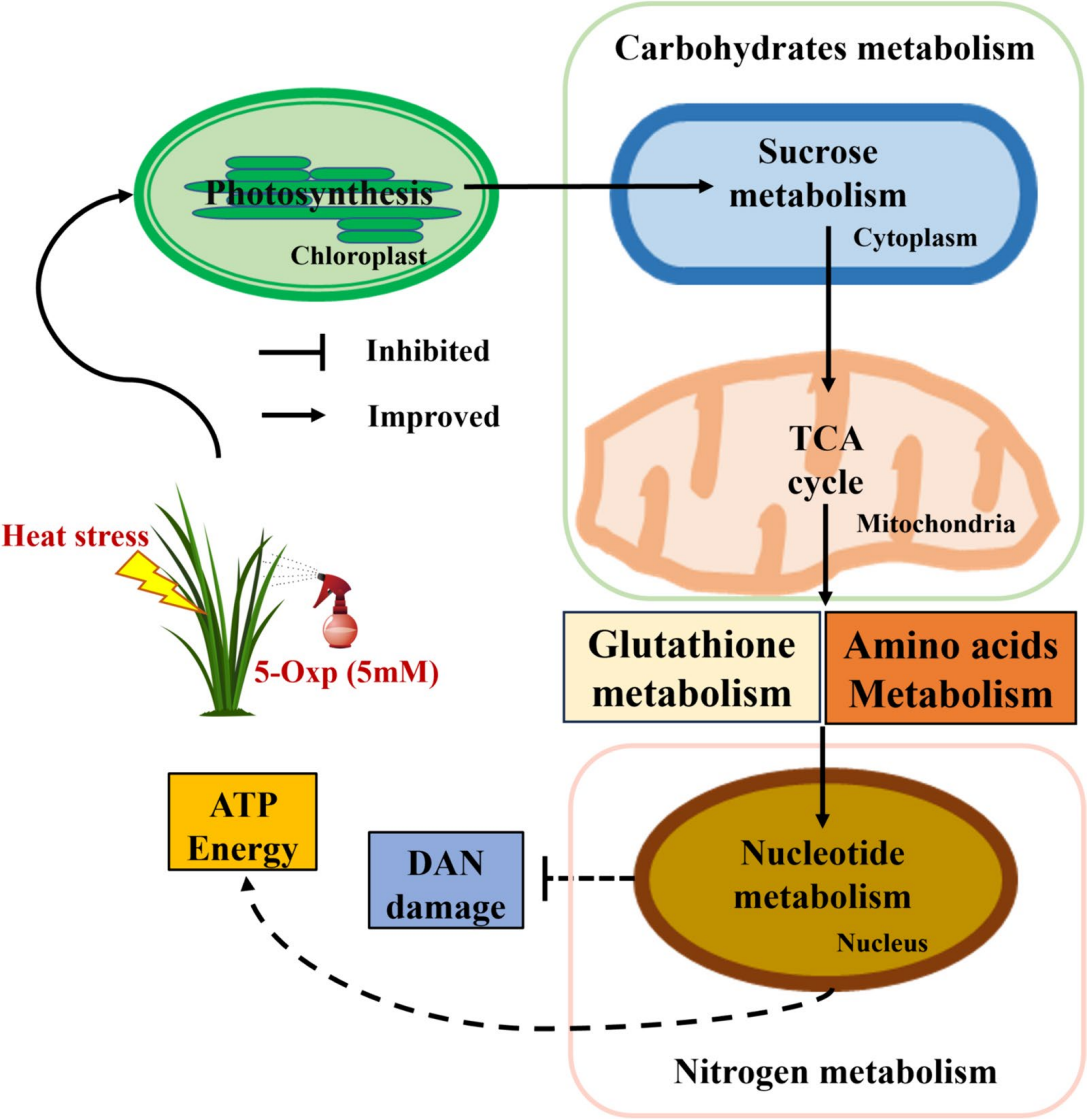
Metabolic regulation of 5-oxp-mediated enhancement in heat tolerance of perennial ryegrass

Heat tolerance mechanisms involve an accumulation of various metabolites, including sugars, amino acids, organic acids, and nucleic acids, which play essential roles in regulating plant growth and development (Hasanuzzaman et al. 2013, Xalxo et al. 2020). This study first demonstrated that with the exogenous application of 5-oxp, the heat tolerance of perennial ryegrass was effectively improved, as manifested by the increase in leaf Chl content, F_v_/F_m_, and antioxidant enzyme activity, as well as the reduction in EL and H_2_O_2_ production under heat stress. The enhanced heat tolerance conferred by 5-oxp was associated with the up-regulation or activation of the metabolism of sugars, amino acids, organic acids, and nucleic acids, with specific metabolites of each functional category discussed in detail below with their associated metabolic pathways (Fig. 8).

Metabolic profiling showed that amino acids were highly responsive to 5-oxp and most were up-regulated only under heat stress (serine, tryptophan, alanine, histidine, methionine, aspartate, asparagine, threonine, proline, and glutamine). The content of two amino acids (glutamate and glycine) associated with glutathione metabolism also increased in response to the application of 5-oxp under non-stress and heat stress conditions, but it increased to a greater extent under heat stress. The increased glutamate content resulting from exogenous application of this metabolite suppressed chlorophyll degradation and activated amino acid metabolism through the pathways of antioxidant defense, nitrogen balance, and energy production in creeping bentgrass (*Agrostis stolonifera* L.) (Rossi et al. 2021). Aspartate, asparagine, threonine, methionine, proline, and glutamine, which are associated with the aspartate and glutamate metabolic pathways, are involved in many biochemical processes, such as the biosynthesis of other amino acids, nucleotide metabolism, the TCA cycle and intermediate glycolysis, and hormone biosynthesis (Han et al. 2021, Newsholme et al. 2003). Previous studies prove that aspartate, methionine, threonine, glutamine, alanine, serine, or tryptophan is significantly accumulated in response to high temperatures, which can enhance the heat tolerance of perennial ryegrass, creeping bentgrass, or tall fescue (Lei and Huang 2022, Lei et al. 2021, Rossi et al. 2021). All of the amino acids up-regulated by 5-oxp, with the exception of proline, are proteinogenic amino acids. Proline, as a non-proteinogenic amino acid, is well-known for its roles in plant adaptation to abiotic stresses, including heat stress, in various plant species and functions during osmotic adjustment for cellular turgor maintenance, the maintenance of membrane stability, and antioxidant protection (Hayat et al. 2012). As discussed in the introduction, 5-oxp serves as a precursor for glutamate, which serves as an ammonia donor for all other amino acids; therefore, 5-oxp could enhance amino acid metabolism, which plays an essential role in regulating plant growth and stress tolerance (Teixeira et al. 2017). The increased availability of 5-oxp through exogenous application could enhance amino acid availability for protein synthesis; however, whether 5-oxp affects downstream protein metabolism and which proteins could be responsive to 5-oxp deserve further investigation.

Application of 5-oxp also caused up-regulation of carbohydrate metabolism in both photosynthesis and respiration in perennial ryegrass exposed to heat stress in this study. Glucose, ribulose 5-phosphate, glucose-6-phosphate, and rhamnose are products of photosynthesis and play critical roles in supplying energy for many biological functions (Atkin et al. 2000). Pyruvate is the final product of glycolysis and the initial organic acid that enters the TCA cycle of respiration, while malate and succinate are key intermediates in the TCA cycle for the generation of reductants and adenosine triphosphate (ATP) (Akram 2014). The accumulation of the carbohydrates and organic acids up-regulated by 5-oxp has been associated with tolerance to heat, cadmium, or drought stress in perennial ryegrass, creeping bentgrass, tall fescue, or bermudagrass (*Cynodon dactylon*) (Jespersen and Huang 2017, Lei and Huang 2022, Lei et al. 2022, Xie et al. 2014, Yu et al. 2012). The results presented in this study suggest that the exogenous application of 5-oxp could stimulate carbohydrate metabolism to maintain energy supply and support heat tolerance in perennial ryegrass.

Nucleotides are building blocks of nucleic acids that control the genetic makeup and energy metabolism of plants (Felig 1975, Zrenner et al. 2006). In this study, the content of four nucleotides (UMP, UDP-glucose, xanthosine, and thiamine) significantly increased in perennial ryegrass treated with 5-oxp under heat stress, all of which related to purine and pyrimidine metabolites in the nucleus that play essential roles in DNA and RNA synthesis, as well as ATP energy synthesis (Zrenner et al. 2006). The rice (*Oryza sativa*) mutant of the UMP kinase gene led to a reduction in Chl content and photosynthetic capacity, while sensitivity to cold stress was increased (Dong et al. 2019). The overexpression of UDP-glucose pyrophosphorylase (UGPase) improved the plant growth and metabolism of tobacco (*Nicotiana tabacum*) (Coleman et al. 2006). Xanthosine regulates the RNA synthesis of *Arabidopsis thaliana* seedlings (Riegler et al. 2011). Thiamine is a fundamental enzymatic cofactor in glycolysis metabolism, and an increase of endogenous or exogenous thiamine content in plants can enhance resistance to abiotic stresses, including salt, oxidation, heat, cold, and drought (Goyer 2010). Therefore, our results illustrate that exogenous 5-oxp positively regulates nucleotide metabolism, which could improve ATP energy generation and promote the maintenance of DNA and RNA synthesis, leading to an improved heat tolerance of perennial ryegrass.

In summary, the exogenous application of 5-oxp promoted heat tolerance of perennial ryegrass. Enhancement of heat tolerance by 5-oxp could be associated with the up-regulation or activation of sugar and organic acid metabolism during photosynthesis and respiration, amino acid metabolism for protein synthesis, and the maintenance of nucleotide metabolism for ATP, DNA, and RNA synthesis (Fig8). This study provides insight into the metabolic regulation of 5-oxp in terms of cool-season grass tolerance to heat stress, although the upstream molecular effect that 5-oxp may have on regulating plant tolerance to heat stress is unknown and deserves further investigation.

## Materials and methods

### Plant materials and growth conditions

Perennial ryegrass (cv. Pinnacle) plants were propagated from tillers of mature plants, which were planted in plastic pots (20 cm diameter, 10 cm length) filled with a mixture of sand and soil (1:1, v/v). Plants were kept in a greenhouse for 60 d at 25/20℃ (day/night) and received natural sunlight with an intensity of 760 µmol·m^2^·s^1^ photosynthetically active radiation (PAR) at the canopy level. In this period, the plants were watered once every two days and fertilized once a week with half-strength Hoagland’s nutrient solution (Hoagland and Arnon, 1950). The plants were trimmed once weekly to maintain a canopy height of 8-9 cm. After 6 days of plant establishment in the greenhouse, the plants were transferred to growth chambers controlled at 25/22 ℃ (day/night), 70 % relative humidity, a light intensity of 650 µmol·m^2^·s^1^, and a photoperiod of 14-h.

### Experimental treatments and design

A preliminary experiment showed that a concentration of 5 mM 5-oxp was most effective in promoting the growth of perennial ryegrass under heat stress, as revealed by the plants treated with this concentration exhibiting less electrolyte loss and higher chlorophyll levels under heat stress (Supplemental figure 1). In this study, a 5 mM solution of 5-oxp was foliar-applied to turf daily for 3 days prior to the exposure of those plants to different temperatures, and subsequent applications were made weekly during the temperature treatments. The untreated plants were sprayed with distilled water (DW) of a volume equal to that for the 5-oxp treatment made at the same application intervals. The temperature treatments included the non-stress control, for which plants were maintained in growth chambers at 25/20 °C (day/night, optimum), and heat stress, where plants were maintained at 35/30 °C (day/night). The plants were fertilized once weekly with half-strength Hoagland’s solution and watered twice daily to ensure that the soil was irrigated to full capacity until water leaked from the bottom of each pot.

The experiment was arranged as a split-plot design, with temperature as the main plots and 5-oxp treatment as the sub-plots. Each treatment involved four replicate pots that were exposed to either the non-stress or heat stress temperature treatment in four growth chambers. During the experimental period, plants were relocated randomly across different growth chambers to minimize the potential effect of variable environmental factors among different chambers.

### Physiological assessment of heat tolerance

Leaf EL was analyzed to evaluate cell membrane stability. Approximately 0.2 g of fresh leaves was added to test tubes containing 20 mL of deionized water and then shaken for 24 h at ambient temperature. The initial conductivity (C_initial_) of the solution was measured with a conductivity meter (model 32; YSI, Yellow Springs, OH). Leaves were then autoclaved at 121 ℃ for 20 min to kill the tissues, which were then placed on a shaker for another 24 h. The maximum conductivity of killed tissues (C_max_) was measured, and leaf percent EL was calculated as C_initial_ / C_max_ x 100 (Blum and Ebercon 1981).

For chlorophyll (Chl) content, fresh leaves (0.1 g) were soaked in 10 mL of dimethyl sulfoxide in the dark for 48 h. The absorbance of Chl extract was recorded as 663 and 645 nm with a spectrophotometer (Thermo Fischer Scientific, Waltham, MA), and leaves were dried in a convection oven (Cole-Parmer, Vernon Hills, IL) at 80 °C for 3 d so that tissue dry weight could be obtained. Leaf Chl was calculated using the formula described by Arnon (1949) and expressed on a dry weight basis.

Leaf F_v_/F_m_ was defined as the ratio of variable fluorescence (F_v_) to maximum fluorescence (F_m_). Chlorophyll fluorescence was measured using a fluorometer (Open FluorCam FC 800-O/2020, Photon Systems Instruments, Brno, Czech Republic) and F_v_/F_m_ was analyzed based on the fluorescence image.

### Production of hydrogen peroxide (H_2_O_2_)

The production of H_2_O_2_ in leaves was visualized using the 3’,3-diaminobenzidine (DAB) method (Thordal‐Christensen et al. 1997). A DAB solution was prepared immediately before leaf-staining to avoid auto-oxidation. The first two fully-expanded leaves of a plant sample were cut with a razor blade and incubated in a 1 mg·mL^-1^ DAB solution (w/v; pH 4.0) in the dark at 21-22 °C for 10-12 h. Staining was ended by soaking the leaves in 90 % warm ethanol until the decolorization of tissues was apparent, with the exception of the deep-brown-colored polymerization product produced during the reaction of DAB with H_2_O_2_ in the leaf cells. The content of H_2_O_2_ was measured and calculated, as described by Velikova et al. (2000).

### Antioxidant enzyme activities

A crude enzyme solution was isolated from leaves by grinding 0.3 g of leaf tissue to powder with liquid nitrogen and homogenizing in 3 mL of cold phosphate-buffered saline (PBS) (50 mM, pH 7.8) containing 1% polyvinylpyrrolidone (PVPP) and 0.2 mM ethylenediaminetetraacetic acid (EDTA). The homogenate was centrifuged at 15,000 g at 4 °C for 20 min, and the supernatant was used for measuring the activity of CAT, GR, APX, DHAR, and MDHAR (Zhang and Kirkham 1996). The activities of CAT, GR, APX, DHAR, and MDHAR were determined by reading the absorbance of the reactions at 240, 470, 290, 340, 265, and 340 nm, respectively. The detailed measurement methods of CAT, GR, APX, DHAR, and MDHAR were described by Cakmak et al. (1993), Dalton et al., (1986), and Nakano and Asada (1981).

### Metabolic analysis

Leaf samples were harvested from plants exposed to 25 days of heat stress and lyophilized in a FreeZone 4.5 benchtop freeze drier (Labconco, Kansas City, MO) until tissue weights were consistent. The samples were ground to a fine powder, and 20 mg of leaf tissue powder was added to a 10 mL centrifuge tube. All samples were loaded into a high-performance liquid chromatography-electrospray ionization-mass spectrometer (HPLC-ESI-MS) system comprised of a Dionex UltiMate 3000 HPLC (Thermo Fischer Scientific, Waltham, MA) and Q Exactive Plus MS (Thermo Fischer Scientific, Waltham, MA). The HPLC was equipped with an XBridge ethylene bridged hybrid (BEH) amide column (2.1 × 150 mm^2^, 2.5 μM particle size, 130 Å pore; Waters Corporation, Milford, MA) coupled with an XBridge BEH Amide XP VanGuard cartridge (2.1 x 5 mm^2^, 2.5 μM particle size, 130 Å pore; Waters Corporation, Milford, MA). The operating temperature of the column was set at 25 °C. In the mobile phase, solvent A was comprised of water/acetonitrile (95:5, v/v) with 20 mM ammonium acetate (NH_3_AC) and 20 mM ammonia (NH_3_OH) at pH 9. Solvent B consisted of acetonitrile/water (80:20, v/v) with 20 mM NH_3_AC and 20 mM NH_3_OH at pH 9. The gradient percentages applied to solvent B over time (min) were as follows: 0, 100%; 3, 100%; 3.2, 90%; 6.2, 90%; 6.5, 80%; 10.5, 80%; 10.7, 70%; 13.5, 70%; 13.7, 45%; 16, 45%; 16.5, 100%. The flow rate of the mobile phase passing through the column was 300 μL/min with an injection volume of 5 μL. Mass spectrometry scans were recorded in both positive and negative ion modes at a resolution of 70,000 at a mass-to-charge ratio (m/z) of 200. The automatic gain control target was set at 3 × 10^6^, and the m/z scan range was 72-1000 in positive or negative ion mode. Metabolite data was acquired through the MAVEN metabolomic analysis software using each labeled isotope fraction (mass accuracy window: 5 ppm). Metabolites were annotated based on the accurate mass and retention time recorded in our in-house metabolite library.

### Statistical analysis

Treatment effects and interactions were examined via a two-way analysis of variance using SPSS 13.0 (SPSS Inc., Chicago, IL). Differences were analyzed with Fisher’s protected least significance test with *p* = 0.05 at a given day under heat stress. Heat maps were plotted using the GraphPad Prism 8 software (GraphPad Software, Boston, MA). Principal component analysis (PCA), metabolic pathway enrichment analysis, and Venn diagram generation were accomplished using the Origin 2019 software (OriginLab Corporation, Northampton, MA).

## Supplementary Information

Supplementary Material 1.

Supplementary Material 2.

Supplementary Material 3.

## Data Availability

All data are published in this paper.

## Abbreviations

5-oxp: Pyroglutamic acid (5-oxoproline)
AMP: Adenosine monophosphate
APX: Ascorbate peroxidase
ATP: Adenosine triphosphate
BEH: Ethylene bridged hybrid
CAT: Catalase
Chl: Chlorophyll
C_initial_: Initial conductivity
C_max_: Maximum conductivity
DAB: 3',3-diaminobenzidine
DHAR: Dehydroascorbate reductase
DW: Distilled water
EDTA: ethylenediaminetetraacetic acid
EL: Electrolyte leakage
F_m_: Maximum fluorescence
F_v_: Variable fluorescence
F_v_/F_m_: Photochemical efficiency
GR: Glutathione reductase
GSH: Glutathione
H_2_O_2_: Hydrogen peroxide
HPLC-ESI-MS: High-performance liquid chromatography-electrospray ionization-mass spectrometer
m/z: Mass-to-charge ratio
MDHAR: Monodehydroascorbate reductase
NH_3_AC: Ammonium acetate
NH_3_OH: Ammonia
PBS: Phosphate-buffered saline
PVPP: polyvinylpyrrolidone
TCA: Tricarboxylic acid
UDP-glucose: Uridine diphosphate-glucose (UDP-glucose)
UGPase: Uridine diphosphate-glucose pyrophosphorylase
UMP: Uridine monophosphate
