# Supplementary material for: Metabolic regulation of 5-oxoproline for enhanced heat tolerance in perennial ryegrass
Source: Stress Biol. 2024 Nov 11;4(1):46. doi: 10.1007/s44154-024-00175-9 (PMC11551090; doi:10.1007/s44154-024-00175-9)
Supplement: Supplementary file 1 — Supplementary Material 1. [file 44154_2024_175_MOESM1_ESM.docx]

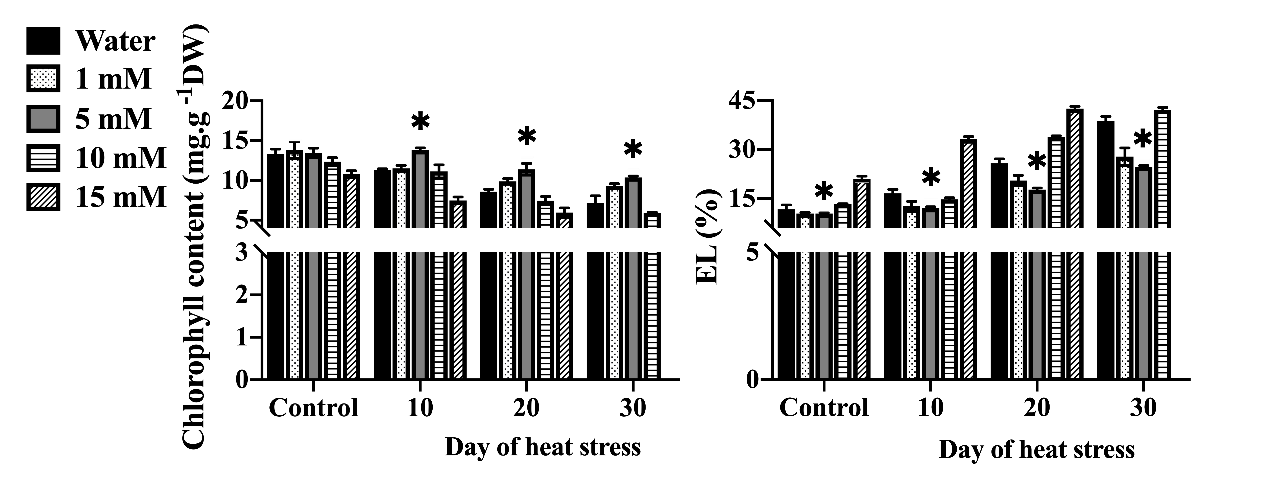


Fig. S1. The effect of water and different concentrations of 5-Oxp on EL and Chl in perennial ryegrass leaves under non-heat stress (control) and 30 d heat stress. Vertical bars stand for standard errors of a certain data point. *: significant differences between DW and 5-Oxp on a certain day of control or heat stress treatment based on the LSD test at p = 0.05 .
