# Supplementary material for: Metabolic regulation of 5-oxoproline for enhanced heat tolerance in perennial ryegrass
Source: Stress Biol. 2024 Nov 11;4(1):46. doi: 10.1007/s44154-024-00175-9 (PMC11551090; doi:10.1007/s44154-024-00175-9)
Supplement: Supplementary file 3 — Supplementary Material 3. [file 44154_2024_175_MOESM3_ESM.docx]

Table 1 The metabolites response to 5-Oxoproline regulating heat tolerance in perennial ryegrass

| No. | RT(min） | Metabolite | Mz | No. | RT(min） | Metabolite | Mz |
| --- | --- | --- | --- | --- | --- | --- | --- |
| 1 | 3.36 | Rhamnose | 163.06 | 30 | 6.48 | Alanine | 88.04 |
| 2 | 12.94 | Ribose-5-phosphate | 232.08 | 31 | 7.01 | Glycine | 74.02 |
| 3 | 9.60 | UDP-Glucose | 565.05 | 32 | 7.49 | Serine | 104.04 |
| 4 | 8.23 | Glucuronic acid | 193.04 | 33 | 7.45 | GABA | 102.06 |
| 5 | 8.23 | Galacturonic acid | 193.04 | 34 | 6.76 | Threonine | 118.05 |
| 6 | 1.99 | Glucuronolactone | 175.03 | 35 | 4.33 | Isoleucine | 130.09 |
| 7 | 10.58 | Glucose 6-phosphate | 259.02 | 36 | 3.88 | Leucine | 130.09 |
| 8 | 7.14 | Sucrose | 341.11 | 37 | 4.73 | Pyroglutamate | 128.04 |
| 9 | 5.77 | Glucose | 179.06 | 38 | 4.94 | Tyrosine | 180.07 |
| 10 | 8.27 | Myo-Inositol | 179.06 | 39 | 4.39 | Tryptophan | 203.08 |
| 11 | 2.54 | Pyruvate | 87.01 | 40 | 9.17 | Histidine | 154.06 |
| 12 | 11.51 | Citrate | 191.02 | 41 | 3.38 | Guanine | 150.04 |
| 13 | 7.34 | Alpha-Ketoglutarate | 145.01 | 42 | 3.04 | Allantoin | 157.04 |
| 14 | 8.79 | Malate | 133.01 | 43 | 2.12 | Uracil | 111.02 |
| 15 | 7.55 | Fumarate | 115.00 | 44 | 2.66 | Adenine | 134.05 |
| 16 | 8.28 | Succinate | 117.02 | 45 | 1.99 | Thymine | 125.04 |
| 17 | 7.05 | Shikimate | 173.05 | 46 | 4.17 | Guanosine | 282.08 |
| 18 | 3.87 | Lactate | 89.02 | 47 | 3.92 | Xanthine | 151.03 |
| 19 | 1.36 | Salicylic acid | 137.02 | 48 | 4.97 | Xanthosine | 283.07 |
| 20 | 5.73 | Ascorbic acid | 175.03 | 49 | 9.40 | AMP | 346.06 |
| 21 | 12.63 | Lysine | 145.10 | 50 | 9.46 | UMP | 323.03 |
| 22 | 7.30 | Glutamine | 145.06 | 51 | 3.90 | Cytidine | 242.08 |
| 23 | 8.48 | Glutamate | 146.05 | 52 | 2.06 | Thymidine | 241.08 |
| 24 | 4.45 | Methionine | 148.04 | 53 | 2.64 | Adenosine | 266.09 |
| 25 | 8.70 | Aspartate | 132.03 | 54 | 6.15 | Thiamine | 263.10 |
| 26 | 7.54 | Asparagine | 131.05 | 55 | 12.25 | GSSG | 611.15 |
| 27 | 3.51 | Phenylalanine | 164.07 | 56 | 8.71 | Glutathione | 306.08 |
| 28 | 5.85 | Proline | 114.06 | 57 | 5.16 | Biotin | 243.08 |
| 29 | 5.38 | Valine | 115.00 |  |  |  |  |
